# Effect of Heavy Metals in Plants of the Genus *Brassica*

**DOI:** 10.3390/ijms160817975

**Published:** 2015-08-04

**Authors:** Miguel P. Mourato, Inês N. Moreira, Inês Leitão, Filipa R. Pinto, Joana R. Sales, Luisa Louro Martins

**Affiliations:** LEAF—Linking Landscape, Environment, Agriculture and Food, Instituto Superior de Agronomia, Universidade de Lisboa, 1349-017 Lisboa, Portugal; E-Mails: ines.nmoreira@gmail.com (I.N.M.); inesibleitao@gmail.com (I.L.); frpinto88@gmail.com (F.R.P.); joanitasales@gmail.com (J.R.S.); luisalouro@isa.ulisboa.pt (L.L.M.)

**Keywords:** *Brassica*, heavy metals, phytoremediation, oxidative stress

## Abstract

Several species from the *Brassica* genus are very important agricultural crops in different parts of the world and are also known to be heavy metal accumulators. There have been a large number of studies regarding the tolerance, uptake and defense mechanism in several of these species, notably *Brassica juncea* and *B. napus*, against the stress induced by heavy metals. Numerous studies have also been published about the capacity of these species to be used for phytoremediation purposes but with mixed results. This review will focus on the latest developments in the study of the uptake capacity, oxidative damage and biochemical and physiological tolerance and defense mechanisms to heavy metal toxicity on six economically important species: *B. juncea*, *B. napus*, *B. oleracea*, *B. carinata*, *B. rapa* and *B. nigra*.

## 1. Introduction

The genus *Brassica* (Brassicaceae) includes more than 30 species plus several varieties and hybrids [1]. Among these are several important species in agriculture, used for human consumption, animal fodder, condiments, oil production, biofuel, among others [2]. Common vegetables used for human consumption are included in this genus, including several *Brassica oleracea* varieties (like cabbages, broccoli, cauliflower and Brussel sprouts). They are considered a source of many nutrients such as carotenoids, tocopherols, different essential elements, carbohydrates and amino acids [3,4].

Several *Brassica* species are known metal accumulators and have been evaluated as potential phytoextraction plants [5,6]. The fact that some of these plants can accumulate relatively high amounts of toxic metals, without visible symptoms, and are also food crops, leads to potential contamination of the food chain [7] and this has to be taken into account in any phytoremediation process. The potential use of *Brassica* species in phytoremediation (mainly phytoextraction) stems from its intrinsic tolerance to heavy metals and considerable above-ground biomass production [8].

This review will focus mainly on six economically important species: *Brassica juncea*, *B. napus*, *B. oleracea*, *B. carinata*, *B. rapa* and *B. nigra* [1,9].

*Brassica juncea* (Indian mustard) is important in oil production, has medicinal properties and is used as a condiment. It is a tolerant plant to heavy metals, grows fast and produces large amount of above-ground biomass. Due to these characteristics, this species has been the target of several studies to evaluate its phytoremediation potential [10]. However, the presence of heavy metals, like cadmium, has been reported to reduce the amount of oil produced by these plants [11].

*Brassica napus* (rapeseed) is consumed as a vegetable but its main use is as a source of oil, being one of the largest sources of edible oil in the world [12]. The by-products from oil production are used in animal feed.

*Brassica oleracea* varieties include very common vegetables used for human consumption, like cabbages, broccoli, cauliflower and Brussel sprouts among others. They are an important source of many nutrients, of compounds with antioxidative activity and of other bioactive compounds such as some glucosinolates that are recognized as beneficial for human health [13,14].

*Brassica carinata* is mainly cultivated in Ethiopia as an oil crop, although it has also received some attention as a source for biofuel production [15].

Both *B. rapa* and *B. nigra* are also used for the oil content of its seeds [9].

There is an increasingly large body of literature describing the effects of heavy metals in different *Brassica* species, confirming the relatively high tolerance that they have towards this abiotic stress, however the exact mechanism for this tolerance remains to be fully clarified [6,16]. In this review, we will evaluate the most recent discoveries in the accumulation and tolerance mechanisms of plants in the *Brassica* genus against heavy metal stress.

## 2. Heavy Metal Uptake and Phytoremediation Potential

Today, large areas all over the world containing arable land are contaminated with heavy metals [17,18]. The pressure to increase crop production can lead to the use of marginal or contaminated soils with a potential danger of food contamination [19]. Some edible plants, include several from the *Brassica* genus under analysis in this review, are known to accumulate relatively large amounts of toxic metals. This has led to the search for plants that can adequately be used for phytoremediation, with the main following characteristics [20]: (i) ability to accumulate the heavy metals in the aboveground parts; (ii) tolerance to the high metal concentrations in soils; (iii) fast growth and high accumulating biomass; (iv) easy to grow as an agricultural crop and easily harvestable.

Most studies regarding the effect of heavy metals on *Brassica* species have focused on a few non-essentials elements (mainly Cd and Pb) and also on some essential elements like Cu and Zn. *B. juncea* is widely regarded as a good plant for phytoremediation purposes and most studies have focused on this species. One of the problems regarding the applicability of plants for phytoremediation purposes is the difficulty in establishing representative experimental setups. Some studies are performed in hydroponic solution, others in soils spiked with heavy metals and other in naturally contaminated soils. Most of the studies reported have been performed under controlled conditions usually in pots, and few in field conditions. The results obtained between all these different experimental conditions are very diverse and it is sometimes difficult to extrapolate the results from one experimental condition to another [21,22]. In Table S1 we present some representative results of heavy metal accumulation in different species under different experimental conditions, but only including contamination by single metals. It is clear the range in concentration of metals in plants of the same species, but of different varieties or cultivars and grown in different experimental conditions. Some studies are performed in hydroponic solution, others in soil spiked with a fixed amount of metals while in others watering is performed with heavy metal contaminated solution. In soil experiments the amount of time between heavy metal spiking and seeding ranges between one week and several months, obviously causing differences in the metal uptake, besides all the large differences in soil characteristics.

Several works have been published comparing the performance of several *Brassica* species to toxic concentrations of heavy metals. Hernandez-Allica *et al.* [23] made an extensive study regarding the heavy metal tolerance of different species (including several varieties of *B. campestris*, *B. rapa*, *B. napus*, *B. oleracea* and *B. carinata*), confirming that they have high levels of tolerance mainly to Zn, and less to Pb and Cd, the metals under study. Purakayastha *et al.* [24] also analyzed the phytoextractive capacity of five different species (*B. juncea*, *B. campestris*, *B. carinata*, *B. napus* and *B. nigra*) to four heavy metals, Zn, Cu, Ni, and Pb. They concluded that *B. carinata* performed better in relation to Zn, Ni and Pb, and would thus be a better candidate for phytoremediation purposes, than the other species, especially *B. juncea*, usually considered very tolerant. In relation to Cu, *B. juncea* presented the highest uptake of this metal. Ebbs and Kochian [25] had previously in 1997 published a study comparing the toxicity and accumulation of Zn and Cu in three species from the genus *Brassica* (*B. juncea*, *B. napus* and *B. rapa*) and concluded that they were more effective at removing Zn than Cu. The apparently different results obtained could be explained by differences in experimental conditions and also of different cultivars used.

On the other hand, Gisbert *et al.* [26] compared the tolerance of three *Brassica* species to a multicontaminated soil and, based on a calculated tolerance index, concluded that the tolerance order was *B. juncea* > *B. carinata* > *B. oleracea*. They also observed that these *Brassica* species behaved as Zn and Pb excluders, able to maintain an almost constant level of these metals in the shoots, up to a certain level of toxicity. In yet another study comparing the phytoextraction behavior of *B. juncea*, *B. napus* and *B. carinata*, Marchiol *et al.* [27] concluded that all these species demonstrated similar potential for both Cd and Zn.

In their study with *B. napus* plants in contaminated soil Brunetti *et al.* [28] reported that the accumulation of different metals under study (Cd, Cr, Cu, Ni, Pb and Zn) was more pronounced in shoots than in roots as is typical of an accumulator species. Curiously Feigle *et al.* [29] observed the exact opposite, as both *B. napus* and *B. juncea* accumulated more Cu in roots than in shoots under Cu stress for 7 and 14 days in hydroponic solution.

These results emphasize that the results observed are highly dependent on the experimental conditions used (some of which are indicated in Table S1), making harder to extrapolate any general conclusions. Naturally the concentrations of the heavy metal under study and the duration of the effect are very important experimental parameters. In addition, not only plant species but also the specific variety/cultivar can have different behavior in regard to heavy metal uptake and effects. Some authors perform the experiments in hydroponics because it is easier to control all other conditions, besides the variable under study. Even so, different hydroponic techniques, nutrient concentrations and pH can also affect plant development. The experiments in soil are usually more complex as the number of variables affecting heavy metal uptake also increase (like soil type, pH, organic matter *etc.*). Some authors use soil contaminated over years of anthropogenic activity while others spike the soil and perform the experiment after a few days or weeks of stabilization and this can have a large impact on the results obtained. Other parameters like the age of the plant at contamination are also critical to the effects usually measured.

Within the same species there is variability in the uptake capacity of different varieties, and even of different accessions [21]. Qadir *et al.* [30] evaluated ten cultivars of *B. juncea* to determine Cd extraction potential. Although the general response to the evaluated parameters was similar, some cultivars presented higher Cd tolerance than others. The uptake values were relatively low (see Table S1) but the maximum duration of the toxicity was 3 days although extremely high concentrations of Cd were used (up to 2000 µM). Gill *et al.* [31] also evaluated the performance of four cultivars of *B. napus* against Cr stress and found significant differences in their tolerance capabilities.

Nouairi *et al.* [32] compared the uptake of Cd between *B. juncea* and *B. napus* and concluded that the first was able to accumulate much higher Cd content in the shoots, mainly at the higher Cd concentrations in hydroponic solution. Seth *et al.* [33] also considered *B. juncea* adequate for phytoremediation not only in relation to Cd contamination but also to Cr, Cu and Pb.

Most of the studies about multielement toxicity, like the ones described above, assume a homogeneous distribution of the metals in soil. Podar *et al.* [34] presented an interesting study with *B. juncea* considering a heterogeneous distribution of the metals, as the authors consider would be closer to what happens in real contaminated sites. The results presented suggest that phytoremediation experiments in homogeneous conditions may underestimate the quantity of contaminants that can be taken up by plants.

As some *Brassica* species are important in oil production, both for human consumption and for the production of biodiesel, the possible contamination of seed and oil from these plants has also been the objective of some studies, as it could lead to contamination of the food chain and of the environment. Park *et al.* [35] studied the suitability of using *B. napus* for phytoremediation purposes and the possibility of using the obtained oil from plants grown in contaminated sites. They reported that the plants could grow and tolerate relatively high levels of a combination of different heavy metals and at the same time produce oil that was safe to be used as an energy source as the content in heavy metals was relatively low (they reported that most of the remaining heavy metals were retained in the residues from the oil extraction process). This could potentially make phytoremediation a more profitable process by linking it to economic valorization. Sankaran and Ebbs [36] devised an interesting experiment, evaluating the toxicity of Cd in *B. juncea* plants and its accumulation in seeds, when applied at different stages in plant development. They concluded that although the levels of Cd in seeds were dependent on the stage of plant development, they could easily accumulate in concentrations above acceptable limits for food crops, limiting its safe consumption.

The increasing demand for water in agriculture has led to the study of alternatives including the use of treated waste waters (TWW) that can be rich in plant nutrients but have also the potential to contain different contaminants including heavy metals. Some recent studies have been made regarding the use of TWW in crop species including some from the genus *Brassica*, showing the dangers of using contaminated waters in the irrigation of plants that can be used for human consumption [37,38].

Other heavy metals have received less attention as they are also of less concern regarding its presence in toxic concentration. For example, Hale *et al.* [39] published an interesting study regarding the toxicity of molybdenum in *B. juncea* and *B. rapa* plants, an essential elements that is needed in very low concentrations by plants but to which they present quite high tolerance for reasons not still fully elucidated.

## 3. Techniques for Improving Heavy Metal Uptake and Tolerance

In order to enhance the availability of heavy metals to allow the effective use of phytoextraction processes, several chemical chelating agents have been proposed to increase the uptake of metals by plants and the translocation from roots to shoots but without affecting plant growth [40]. Several studies have been performed in *Brassica* species, with different chelating agents [18].

The addition of citric acid to the contaminated medium led to increased uptake and amelioration of *B. napus* in relation to Pb [41] and to Cd contamination [42]. This was attributed by the authors of the first study to the improvement of several metabolic processes through the mobilization of essential nutrients, although the exact mechanism for this is still unknown. In two publications Quartacci *et al.* [43] and Irtelli and Navari-Izzo [44] evaluated the influence of adding also citric acid and sodium nitrilotriacetate (NTA) to contaminated soil, in *B. juncea* plants growing under Cd stress. According to their results, the plants were able to accumulate more Cd with the addition of NTA while for citric acid the differences in relation to the control were very small. In a follow-up study by the same group [45] they reported that (*S*,*S*)-ethylenediamine-*N*,*N*′-disuccinic acid (EDDS) was even more effective than NTA in facilitating the phytoextraction of a multi-contaminated soil, this time with *B. carinata* species. This EDTA-isomer had the added advantage of a rapid degradation in the soil, reducing the potential leaching into ground waters. While EDTA itself has also been reported to increase the uptake of heavy metals from contaminated soils by *Brassica* species [46,47,48], there are concerns regarding the environmental persistence of this chelating agent and its strong chelating capacity [49].

Another strategy to improve soils contaminated with heavy metal is the use of organic amendments to reduce the bioavailability of the metals to the plants and/or to improve plant growth. Clemente *et al.* [50] evaluated the practical use of *B. juncea* for phytoextraction at a multi-contaminated site (mainly contaminated with Zn, Cu and Pb), with and without added amendments (cow manure and compost). They concluded that plant growth and metal uptake was highly dependent on soil pH, although the added amendments improved plant growth. They also calculated that between a minimum of 1150 years for Cu and a maximum of 360,000 years for Pb would be necessary for the effective clean-up using *B. juncea* as a phytoextractor obviously making this technique completely unsuitable. Similar results were obtained in the study of Brunetti *et al.* [28] with *B. napus* plants where they reported that the addition of municipal solid waste compost improved the extraction of heavy metals by those plants, however they calculated that more than 1000 years would be necessary to clean up a contaminated site, also making this plant unsuitable for phytoextraction purposes. Other studies confirm that it would take hundreds or thousands of years for the complete clean-up of heavily contaminated sites [51]. These studies highlight the practical difficulties of using phytoextraction as a phytoremediation method for contaminated soils and alternative techniques like phytostabilization or phytoimmobilization might be more effective remediation techniques. In addition, although most experimental studies are done with single contaminants, many practical cases of soil contamination by heavy metals involve multiple metals and this further complicates the study of plant uptake and tolerance mechanisms. Marchiol *et al.* [20] studied the behavior of canola (*B. napus*) in a multi-contaminated soil and concluded that although it presented moderate tolerance to the heavy metals in the soil (including high levels of Cd, Cr, Cu, Ni, Pb and Zn), it was not adequate for phytoremediation purposes. Possibly the combined toxicity of all these metals overcome the defense and tolerance mechanisms of the plant. In their study with *B. napus* var. oleifera, Herrero *et al.* [52] confirmed the relatively high tolerance of this plant to another multi-contaminated soil (Zn, Cu, Cd and Pb).

Houben *et al.* [53] tested the effect of the application of biochar in *B. napus* plants growing in soils contaminated with Zn, Cd and Pb. Biochar is reported to reduce the availability of heavy metals to plants and to increases the soil quality allowing improved plant growth. The authors confirmed that the addition of 5% and 10% biochar to the soil reduced the uptake of the metals and significantly improved rapeseed growth compared to the control. These results lead to the conclusion that while the addition of biochar makes unfeasible the use of rapeseed for phytoextraction due to the reduced metal uptake, it could be an effective strategy for phytostabilization.

Other strategies to improve the phytoremediation capacity of *Brassica* species include the inoculation of plants with certain bacteria with the aim to promote the growth of the plant in a contaminated medium and thus improving the metal uptake capacity. Adediran *et al.* [54] have shown this to be a promising strategy in the phytoremediation of Zn with *B. juncea*.

## 4. Heavy Metal Transporters

Heavy metal transporters are very important in the regulation of metal transport and accumulation in plants, with consequences it their tolerance, and have thus been studied in different plant species including from the *Brassica* family [55]. Bhuiyan *et al.* [10] generated a transgenic *B. juncea* plant, with the introduction of the *AtATM3* gene, a member of the ATP-binding cassette (ABC) transporter family localized in the mitochondrial membrane of *Arabidopsis thaliana*. The overexpression of this gene not only improved the tolerance of the transgenic plant to Cd and Pb toxicity (150 and 1000 µM, respectively) but also increased the translocation of the metals to the shoots, by a factor of between 1.5 and 2.5 compared to the wildtype, thus improving the potential of these species for phytoremediation purposes. Although the mechanism by which these *AtATM3* overexpressing plants presented increased resistance to the tested metals toxicity is not fully understood, the authors postulate that they produce more mitochondrial Fe–S clusters that are transported to the cytoplasm and can counter the induced oxidative stress. Another explanation given is the increase in the induction of metal transporters involved in vacuolar sequestration and the increase in the expression of genes that are involved in the synthesis of glutathione and phytochelatins (see below). This is maybe a more logical explanation as these peptides are known to participate in the defense mechanisms plants use against heavy metal toxicity [56].

In a similar study [57], the same group analyzed the effect of the introduction of yeast cadmium factor 1 (*YCF1*) in *B. juncea* plants, another member of the ABC transporters, like *AtATM3*. They also reported increased accumulation and tolerance of the transgenic plants to Cd and Pb and the main cause was attributed to the role that *YCF1* plays in the transport of the metals conjugated to glutathione from cytoplasm to vacuoles.

Members of the YSL (yellow stripe-like) family have been involved in the transport of different essential elements in plants [58]. This family of transporters might also have a role in the tolerance of plants to heavy metal toxicity. Wang *et al.* [59] isolated a *B. juncea* YSL gene, *BjYSL7*, whose overexpression increased heavy metal tolerance of tobacco plants and this, according to the authors, was due to the increased transport of the metals (Cd and Ni) from roots to shoots, preventing damage to the roots.

Some studies have also been made regarding the role of cation-efflux family transporters (CET) in *Brassica* plants tolerance to heavy metals. Xu *et al.* [60] reported that the overexpression of *BjCET2* conferred heavy metal tolerance and increased Cd/Zn accumulation in the leaves of transgenic *B. juncea* plants, confirming the importance of these transporters in the translocation of heavy metals in the plants. Lang *et al.* [61] reported that two other members of the same family, *BjCET3* and *BjCET4*, may be involved in regulating the homeostasis of several heavy metals like Cd, Co and Ni in *B. juncea*. MTP1 is another type of cation-efflux proteins and Muthukumar *et al.* [62] reported enhanced mRNA levels of *BjMTP1* in *B. juncea* plants under Ni and Cd toxicity (and much less under Zn toxicity). The authors argue that while there is no evidence to suggest that *BjMTP1* is used in the transport Cd or Ni, it might have a function as a regulator of Zn homeostasis as part of the plant response to the toxic effects of the other two heavy metals.

There thus seem to be some clear possibilities to increase the phytoremediation potential of some *Brassica* species by using certain transgenic plants and patents have already been registered to this effect [63].

Other genetic changes increase plant tolerance by limiting metal uptake, and this could be useful to develop edible plants that could be grown in contaminated sites that would otherwise be unsuitable for crop production. Li *et al.* [64] developed transgenic *B. juncea* plants overexpressing *heme oxygenase-1* (*HO-1*), a stress response gene coding for an enzyme involved in the heme catabolism but whose role in metal tolerance is not clear. In this work, it was confirmed that these transgenic plants accumulated less Hg and showed reduced Hg-induced oxidative stress.

## 5. Physiological Damage

The damage induced by toxic amounts of heavy metals has been attributed to different causes that usually act together, and can include direct metal damage and indirectly via induced oxidative stress [65]. Usually reported effects include reduction in chlorophyll (either to decreased synthesis or increased degradation), altered water balance, decreases activity of various enzymes, stomatal closure, slowing down of photosynthesis rate and reduced uptake of essential mineral nutrients [17].

Several studies with heavy metals report a decrease in the water content in heavy metal stressed plants, and so, the toxic effect of metals could be caused indirectly, by a reduced water uptake [66]. For instance, Zaier *et al.* [67] reported that the water content of *B. juncea* plants drastically decreased under Pb toxicity, even in this species that is considered tolerant. Some authors have reported that soluble sugars, proline and other amino acids can have a protective role, regulating osmotic potential and also with a more direct detoxification role of reactive oxygen species (ROS) [68,69,70]. Ali *et al.* [66] confirmed this protective role in *B. napus* plants under Cd stress, as the foliar application of 5-aminolevulinic acid led to improved plant resistance and to an increase in the levels of these substances. It should be noted however that this study was done under very high Cd concentrations (100 and 500 µM) and that both proline and soluble sugars decreased in Cd contaminated plants probably due to general damage in the plant metabolism.

Usually the first visible symptoms related to heavy metal toxicity include not only stunted growth, but also reduced root growth and changes in root morphology [29]. Normally roots are more affected than leaves as they are the organs in direct contact with the toxic element. Most studies of species from the genus *Brassica* report reduced root growth under different heavy metals [25,71]. However, different metals can exhibit different effects. Ebbs and Kochian [25] reported that Cu had a more pronounced effect in lateral root development than Zn. Nouairi *et al.* [32] reported also a reduction in both root and shoot growth in *B. juncea* and *B. napus* under Cd stress, and hypothesized that this could be due to restriction in the uptake of Fe and Mn to the shoots, but presented no data to support it.

Barrameda-Medina *et al.* [72] reported reduced root biomass of *B. oleracea* plants under Zn toxicity (500 µM) but no changes to foliar biomass, because Zn, as other heavy metals, mostly accumulate in the roots. In *B. napus* plants, Ghnaya *et al.* [73] detected a reduced biomass both under Zn and Cd stress, although at higher concentrations (2000 and 250 µM respectively), except for one of the four cultivars under study. A similar effect was detected in *B. rapa* plants growing under Zn toxicity, at a concentration of 500 µM [74].

In a study about the toxicity of Cu on *B. juncea* plants, Wang *et al.* [75] detected a significant decrease in root biomass at Cu concentrations as low as 4 µM. On the same species but in a study with Pb, Zaier *et al.* [67] reported decreased plant biomass with Pb concentrations of 200 µM and higher. Concentrations of 50 and 100 µM of lead have been reported to significantly affect *B. napus* plants growing in hydroponic solution [41]. Growth parameters, chlorophyll and carotenoids content were significantly affected and both hydrogen peroxide and malondialdeyde levels increased significantly indicating the occurrence of oxidative stress.

Although Cd is highly toxic to plants [76], both Armas *et al.* [22] and Seth *et al.* [77] reported an early increase in growth parameters of *B. juncea* plants growing under Cd-contaminated soil and in hydroponics, respectively, and the authors attribute this to a hormetic effect that could be due to an “overcompensation” response to a disruption in the plant homeostasis. At later exposition times this effect was reverted, and toxicity effects were noticeable. Of note is the fact that Armas *et al.* [22] only reported this in the soil experiment and not in hydroponics as described in the same publication.

Different plants are able to translocate heavy metals to other plant parts, partially as a defense mechanism. Nouairi *et al.* [32] reported that Cd accumulated in the trichomes of *B. juncea* leaves, and this could in part explain the tolerance of these species to heavy metals as the trichomes are an external tissue. A similar conclusion in the same species had been reached in 1995 by Salt *et al.* [78]. However the mechanisms that would allow this are not fully clear.

Seed germination can also be affected by heavy metals and Siddiqui *et al.* [79] reported that *B. rapa* var. turnip was more affected by Cd, followed by Cr and by Pb.

Most metals induced oxidative stress, either directly, like Cu, via Haber-Weiss and Fenton reactions or indirectly by causing imbalances in the anti-oxidative system [80]. The direct effects of this are usually measured via the levels of hydrogen peroxide (a ROS that is easily quantified) and malondialdehyde (MDA), a product of ROS-induced lipid peroxidation. All the different ROS, including hydrogen peroxide, are not only a toxic product resulting from oxidative stress [81] but are now known to be important signaling molecules that have been implicated in numerous responses against different abiotic stresses [82,83].

The direct measurement of ROS is notoriously difficult, in part due to its high reactivity and short life-span. Most experiments measure the levels of H_2_O_2_ and use this value as indicative of the presence of ROS in the plants, and usually when other ROS are measured, like O_2_·^−^, an increase in their content is also detected under heavy metal toxicity [31] although sometimes results are more variable [29]. The increase in H_2_O_2_ levels is frequently reported in relation to the toxicity of different metals [31,41,42,74,84], although other studies have also reported a decrease in their contents [29,85].

The increase in MDA content is a frequently described effect of heavy metal toxicity in different *Brassica* plants [74,84]. Ghnaya *et al.* [73] also reported an increase in MDA levels due to Zn and Cd toxicity in *B. napus* plants and further concludes that the chloroplast membrane is particularly sensitive to the ROS action due to the high contents of polyunsaturated fatty acids, added to the fact that most ROS should accumulate in the thylakoidal membranes resulting from photosynthesis. In their comparative study of *B. napus* and *B. juncea* under Cd stress Nouairi *et al.* [32], reported a dose-dependent reduction in the lipid content of *B. napus* but not of *B. juncea* plants. A reduction in the ratio of monogalactosyldiacylglycerol (MGDG) to digalactosyldiacylglycerol (DGDG) was detected which could mean a loss of PSII complex stability. In addition, Cd preferentially affected chloroplast lipids containing higher levels of polyunsaturated fatty acids. In *B. juncea* plants, an increase in membrane lipids was detected, which could lead to an increase in the vacuolar area where toxic metals are usually stored. Generally cellular membranes of *B. juncea* were not as affected by Cd and this could justify the higher tolerance of this species.

Most of the studies related to oxidative stress in *Brassica* species are performed in adult plants, although with plants at different ages. Szollosi *et al.* [86] in a study of Cd toxicity in *B. juncea* seeds confirmed that they absorbed considerable amounts of this heavy metal and that oxidative stress occurs very early in plant development.

The presence of heavy metals in toxic amounts is known to affect photosynthetic activity [87]. A reduction in photosynthetic pigments, and the appearance of chlorosis effects, is a frequently described effect of heavy metals in plants, either due to reduced chlorophyll synthesis (caused by the inhibition of the respective enzymes), increased chlorophyll destruction or affected element uptake (either by inhibition of the uptake or by competition with other heavy metals). Other causes for affected photosynthesis function include the inhibition of the Calvin cycle [66], reducing CO_2_ fixation, the reduced aggregation of pigment protein complexes of the photosystems and general ROS induced damage to the chloroplasts [32,84]. Most of the effects of heavy metals in chlorophyll are based on the measurement of the contents of this pigment and this leads to most authors justifying its decrease due to the inhibition of the enzymes responsible for its synthesis, although this conclusion is usually based on indirect evidence.

The ratio between chlorophyll *a* and chlorophyll *b* is also an important parameter and its increase have been associated to lower levels of light harvesting chlorophyll proteins (LHCPs) [84].

Both Ali *et al.* [66] in *B. napus* and Ahmad *et al.* [11] in *B. juncea* plants reported a reduced photosynthesis induced by Cd stress, which the authors attribute either to inhibition of the synthesis of important enzymes in the formation of chlorophyll or to the impairment (or competition) in the supply of important elements, like Fe, Mg, Zn and Mn (see below). Contrary to these findings Baryla *et al.* [88], in their study of *B. napus* growing in Cd contaminated soil, also reported leaf chlorosis but suggested that this was not due to inhibition of chlorophyll biosynthesis nor to mineral deficiency or oxidative stress but to a Cd interference with cell division and chloroplast replication leading to a reduction in the number of chloroplasts per cell.

Ghnaya *et al.* [73] also reported reduced chlorophyll and carotenoid contents under Zn and Cd stress in *B. napus* plants, and justified the reduction due to the inhibition of enzymes leading to chlorophyll synthesis and the reduction in carotenoids due to the increased production of ROS. The chlorosis induced by Zn has often been attributed to an interference with Fe metabolism, although it can also be due to Mn deficiency. However Ebbs and Kochian reported in their 1997 study [25] decreased Fe and Mn content in shoots but little or no reduction in chlorophyll content.

Mobin and Khan [84] studied in detail the effect of Cd on the photosynthetic activity of two *B. juncea* cultivars and found differences between them highlighting the difficulty in making generalizations as different cultivars might respond differently to heavy metal stress. They confirmed that Cd affected the activity of Rubisco and of carbonic anhydrase. The contents of anthocyanins were also found to increase and this could also be part of the defense mechanism to protect the photosynthetic apparatus. In fact, Hale *et al.* [39] proposed that anthocyanins might have an active protective role in *Brassica* plants against Mo toxicity by complexing this metal and sequestering it into the vacuoles.

In contrast to the more common reports with other metals, Gupta *et al.* [89] indicated that chlorophyll (as well as carotenoid) content actually increased in *B. juncea* plants under Cr stress (as Cr(VI)), but this was explained as chlorophyll was measured per mass unit and plants grew significantly less in the toxic environment. However, they also measured PSII activity and it was shown to increase up to 400 µM of Cr and this was attributed to Cr-induced stabilization of the oxygen evolving complex. In another study, with Cr, Gill *et al.* [31] reported a decrease in the net photosynthetic rate in different *B. napus* cultivars under Cr toxicity, especially at the highest studied concentration of 400 µM.

## 6. Micronutrient Status

The presence of excessive amounts of heavy metals (either essential or non-essential) can affect the uptake of other essential elements, but results are very diverse and no clear trend can be established. Ebbs and Kochian [25] reported a general decrease in Mn and Fe when *B. napus* and *B. rapa* plants were subjected to Cu, Zn and Cu + Zn toxicity (with less clear effects for *B. juncea*). However they reported an increase in the levels of these elements in the roots and this confirms that what was affected was the translocation from the roots to the shoots and not the uptake by the roots. According to Zaier *et al.* [67] more tolerant plants are able to selectively absorb essential nutrients and maintain adequate nutrition of their organs, and they report a significant reduction in nutrients concentration in *B. juncea* under Pb stress. In a study about the toxicity of Cd in *B. juncea* plants grown in Cd-contaminated soil, Sikka and Nayyar [90] detected significant reductions in the uptake of Fe and also, to a lesser degree, of Cu and Zn. In *B. napus* under Cd stress [88], only Zn levels were significantly reduced in the leaves but not below the deficiency threshold. In a hydroponic experiment with *B. juncea* and *B. napus* plants with excess Cu, Feigl *et al.* [29] detected a significant reduction in the contents of Zn, Fe, Mn and Co, but the effect was not dose dependent.

As described above, the lack of some of these nutrients could partly explain some of the negative effects usually detected in the photosynthetic apparatus and the appearance of chlorosis symptoms [29], although this is certainly not the only cause for affected photosynthesis.

Besides the differential effects that heavy metal toxicity has in the uptake of essential elements as described above, the interaction between these elements further complicates the issue. Some studies have also reported that the addition of an essential element can reduce the toxic effect of other heavy metals [91]. Podar *et al.* [34] reported that extra Zn could supress Cd uptake in *B. juncea* plants growing in Cd-contaminated soil, compared to the control with regular amounts of the essential element.

## 7. Enzymatic Defense Mechanisms

As referred above, heavy metals generally induce oxidative stress in the plants and affect several metabolic processes [81], although the exact mechanisms by which different heavy metals are toxic to plants are still not fully clear. Plants have an array of different layers of defense mechanisms that reduce the levels of heavy metal toxicity, and the tolerance capacity of each plant is dependent of the cooperative function of all these different mechanisms that include the induction of both enzymatic and non-enzymatic substances [92]. The enzymatic mechanisms include several enzymes that work together to avoid the deleterious effects of ROS and other toxic species. In Table S2 we present selected results of the reported activities of enzymes involved in the antioxidative stress defense mechanisms. Looking at this table it is easy to see that there is no clear behavior of most of the enzymes, as the activities are highly dependent on a large range of experimental factors (as described previously in relation to heavy metal uptake), although there are some trends that can be established.

Superoxide dismutase (SOD, EC 1.15.1.1), is usually considered to be the first line of defense against oxidative stress, converting O_2_·^−^ species into H_2_O_2_ that can then be converted to water by peroxidases and catalase [92]. From Table S2 we can see that there is a general trend for the increase in SOD activity in different *Brassica* species and different metals, although there are some exceptions, but the subsequent decomposition of hydrogen peroxide to water seems to be more genera and metal dependent.

Both ascorbate peroxidase (APX, EC 1.11.1.11), catalase (CAT, EC 1.11.1.6) and guaiacol peroxidase (GPOD, EC 1.11.1.7) catalyse the decomposition of H_2_O_2_ to water, although at different rates with different affinities and in different organelles [81] and the activities of these enzymes have been studied in numerous experiments with *Brassica* species and different heavy metals, as can be seen in Table S2. Generally most authors report increases in these enzymes activities although in certain cases a decrease in the activity is reported. Therefore, the exact mechanisms through which plants get rid of excess hydrogen peroxide is highly dependent not only of the species but on all the different exogenous factors affecting the experiment although at least one of these enzymes is usually found to increase its activity.

In *B. juncea* under Cu stress Wang *et al.* [75] reported in increase in APX and GPOD and a decrease in CAT, while in the same species John *et al.* [71] also reported increase in SOD and APX and a decrease in CAT, with increasing Cd and Pb toxicity. In this last experiment the induced toxicity lasted for a long time (up to 60 days) and a general decrease in enzyme activity was also observed for the longer periods and higher concentrations (300 mg/kg Cd and 500 mg/kg Pb). In contrast to these observations Minglin *et al.* [93] detected an increase in the transcript amount of the *catalase 3* gene (*CAT3*) also in *B. juncea* (under Cd stress), although the enzyme activity was not directly measured. Nouairi *et al.* [94] also detected an increase in CAT activity with increased Cd concentration in *B. juncea* plants, although the effect in *B. napus* was exactly the opposite. In a study of Cr toxicity on *B. juncea* and *B. napus* plants [95], CAT activity decreased and APX activity increased only transiently.

The enhanced GPOD activity in roots reported in some studies [22,96] could also be related to increased lignin synthesis, that could have a putative protective role resulting in cell wall stiffening.

Ascorbate and glutathione are also an important component of plant antioxidant defense as they are part of the ascorbate-glutathione cycle, which is an important metabolic pathway for the removal of ROS. Both glutathione and ascorbate themselves are powerful antioxidants and glutathione is necessary for the synthesis of phytochelatins, a family of important sequesters for heavy metals [97]. Ascorbate increase as a result of induced heavy metal stress is a commonly described plant response, including *Brassica* species, and this could implicate its action as an antioxidant acting as part of the plant defense system [29], although a reduction in ascorbate contents with increasing metal concentration has also been reported [30].

In *B. juncea* plants, several enzymes involved in the ascorbate-glutathione cycle (like APX and glutathione reductase, GR, EC 1.6.4.2) have been reported to increase its activity under heavy metal stress confirming the importance of this metabolic pathway in conferring resistance to this type of abiotic stress [11,66,84], although the results are not always concordant [94]. GR is important in the regeneration both of the reduced glutathione and of reduced ascorbate, that were previously oxidized by H_2_O_2_ [92].

Ali *et al.* [66] also detected an increase in all the studied enzymes at a concentration of 100 µM Cd (Table S2) although a decrease was measured at the very high concentration of 500 µM, which is probably due to general damage to the plant at this stage. Interestingly, they also analyzed the expression of the same enzyme genes that confirmed the effect observed by measuring their activities. Seth *et al.* [77] observed a similar effect in the same species regarding GR activity, although the decrease at higher concentrations was only detected at the longer exposition time of 28 days, as for 14 days after induced toxicity GR activity always increased with Cd up to the maximum studied concentration of 160 µM.

A decrease in enzymatic activities at higher concentrations or at extended periods of time after contamination is also frequently described, indicating that the toxic effects have probably overcome the antioxidant defense capacity of the plant. Actually, while the increase in the activity of antioxidative defense enzymes can logically be attributed to their induction due to the heavy metal toxicity, its decrease has also been justified by the disruption of the anti-oxidative mechanisms due to the same toxicity. However the threshold levels between both effects are very difficult to determine because they are dependent on a large number of factors.

Most studies regarding the effect of metal toxicity report quantitative results for anti-oxidative enzymes and there are fewer studies with more specific gene expression measurements [98]. Although the upregulation of a gene might not directly translate in higher enzyme activities, the enzyme activity measurements are not as specific as they usually measure global enzyme activities. For example, SOD has three isoforms, FeSOD (present in the chloroplasts), MnSOD (present in mitochondria and peroxisomes) and Cu/ZnSOD (present in chloroplast and cytosol) and these are encoded by an even higher number of genes [99].

## 8. Heavy-Metal Chelating and Other Effects

Jahangir *et al.* [100] studied the accumulation of different metabolites in *B. rapa* plants under Cu, Fe and Mn toxicity. They detected an increase in the contents of amino acids, phenolics and glucosinolates, with Cu and Fe toxicity and as the first two are known to have a metal-chelating effect this might be a response to the metal toxicity.

Organic acids are other molecules that have been reported to have a role in the protection against heavy metal stress [101]. Contrary to other effects, an increase in the levels of citrate has been almost universally described as a response to different metals in *Brassica* species. In *B. oleracea* plants growing under Zn stress, Barrameda-Medina *et al.* [72] justified the tolerance of the leaves to this stress to an increase in the levels of malate (that would facilitate the transport of Zn to the leaves) and to the maintenance of high levels of citrate (that would complex the metal in order to sequester it to the vacuole or simply to protect the cell from free Zn). However no increase in the levels of citrate were measured compared to the control. On another study about Zn toxicity in *B. rapa*, Blasco *et al.* [74] detected significant increases in the levels of citrate which could justify the results obtained in the other experiment, and so it seems that this organic acid is important in maintaining metal ion homeostasis. On the other hand they detected a decrease in the concentration of malate and no changes in oxalate levels. Another recent confirmation of the role of citrate in the tolerance to heavy metals was published by Ghnaya *et al.* [102] in the response of *B. juncea* to Pb, where it was reported to have an important role in lead translocation and shoot accumulation, as its levels increased both in xylem sap and in shoots. Irtelli and Navari-Izzo [44] reported an increase in total organic acids in response to Cd toxicity in *B. juncea* plants, confirming the importance of these compounds as a response mechanism. In the same study they also detected an increase in phenolic acids that could have a similar protective role, as they can also act as metal chelators.

Phytochelatines (PC) are small peptides with the general structure (γ-Glu–Cys)*_n_*–Gly, with *n* usually ranging from 2 to 11, that participate in the defense against heavy metal toxicity, by sequestering metal ions into the vacuoles [56]. Its synthesis is catalyzed by phytochelatin synthase (PCS) using glutathione (GSH) as a substrate. Gasic and Korban [103] developed a transgenic *B. juncea* expressing an *Arabidopsis thaliana AtPCS1* gene, that encodes PCS. They observed that low *AtPCS1*-expressing lines showed increased tolerance to Cd and Zn but did not improve the accumulation of these metals. However the increased tolerance to Cd was only confirmed for the concentration of 100 µM (in itself a high concentration) as for higher concentrations, no differences to WT were detected. According to the authors, this could be due to the toxicity of excess levels of phytochelatins or to other type of toxicity in the processing of Cd-PC complexes. On the other hand, the increased formation of PC depleted the pool of GSH, which could limit the tolerance of the transgenic plants. In this same study, no PC were detected in control conditions confirming that this gene is regulated post-translationally and is only active when excess heavy metals are present. Nouairi *et al.* [94] reported a decrease in GSH levels in *B. juncea* plants under Cd stress which the authors attributed to increased levels of PC synthesis (quantified in this study as the difference between non-protein thiols and GSH contents), although this increase in PC content was only detected for *B. juncea* and not for *B. napus* plants. Gadapi and Macfie [104] also compared Cd stress effects on *B. juncea* and *B. napus*. According to their results, *B. juncea* accumulated more PCs in the roots while *B. napus* had more in the leaves. However, the differences in the induced stress effects in both species were reported to be minimal. Thus, the authors concluded that other factors, besides PC concentrations, must exist to explain the observed differences like the production and utilization of cysteine or the ability to transport Cd–PC complexes to the vacuole.

Seth *et al.* [33] reported an increase in GSH contents for lower concentrations of Cd in *B. juncea* plants, followed by a decrease at higher concentrations, and this effect was more pronounced for longer exposition times. The reasons given for this decrease were the same as reported above to which the authors added the possibility that a putative H_2_O_2_ accumulation (not measured in this study) could have caused a down-regulation of genes encoding mRNA for GR involved in GSH synthesis. On the other hand, Schaëfer *et al.* [105] had much earlier, in 1997, reported an increase in the transcript amounts of γ-glutamylcysteine synthetase (γ-ECS), an enzyme which catalyses the first step of the GSH synthesis, in *B. juncea* plants under Cu stress but not under Cd stress, indicating a differential metal-type effect in relation to GSH synthesis and the authors also explained this by a dependence on plant development stage.

Several other studies have reported the induction of PC synthesis under heavy metal stress, confirming that this is an important tolerance strategy that plants from the *Brassica* genus use. Saathoff *et al.* [106] confirmed a time and dose-dependent increase in PC levels in *B. napus* plants after 24 h of Cd induced toxicity, even at low Cd levels, while Zaier *et al.* [67] detected with a certain degree of confidence that PC are involved in the binding and transportation of Pb in *B. juncea* plants. Minglin *et al.* [93] identified a number of genes upregulated in *B. juncea* under sub-lethal concentrations of Cd, which included some involved in GSH synthesis and sulphur assimilation confirming the important role PC have as a detoxification mechanism in these plants.

After extended periods of contamination or higher concentrations of toxic elements, usually a decrease in PC levels can occur probably due to depletion of the GSH pool, declined GR activity or inactivation of PCS enzyme [77].

Metallothioneins (MT) are low molecular weight cysteine-rich peptides that have been recognized to have an important role in the detoxification of heavy metals in plants [107]. In 1997 Schaëfer *et al.* [105] reported no increase in transcript amounts for MT2 in *B. juncea* plants under Cd and Cu stress indicating that MT were not relevant in the tolerance of this species to heavy metals. In order to clarify the role of these peptides Ahn *et al.* [108] analyzed the effect of different heavy metals in the expression of three *B. rapa* MT genes (*BrMT1-3*). However, the authors only studied essential elements (Fe, Cu, Zn and Mn) and no clear and consistent behavior could be established as the expression of these three genes was highly dependent on the metals studied and on the time period under consideration, although the *BrMT1* was the gene whose expression was more upregulated. These results indicate that *Brassica* species probably favor other mechanisms rather than MT synthesis for heavy metal tolerance.

MicroRNAs (miRNAs) are small non-coding RNA molecules that have a function in RNA silencing and post-transcriptional regulation of gene expression. They are known to be involved in the response of plants to several environmental stresses including heavy metal toxicity [109]. Several recent studies have been made to try to unravel the role of miRNA in the tolerance of *Brassica* species against heavy metal stresses (mainly studying Cd). Zhou *et al.* [110] showed that the expression of some miRNAs that could be involved in *B. napus* tolerance to Cd, were differentially regulated by Cd exposure. In another Cd study Zhang *et al.* [111] showed that transgenic *B. napus* plants over-expressing miR395 demonstrated a lower degree of Cd induced oxidative stress than the wild type. An accumulation of sulphur in the plant and a higher content of non-protein thiols (including PCs) was detected in the transgenic plants which could partially explain the higher tolerance of these plants to Cd. This was confirmed by the detection of a higher expression of the gene *BnPCS1* involved in PC synthesis.

To overcome some of these known toxic effects of heavy metals, namely Cd, Hayat *et al.* [112] reported that *B. juncea* plants sprayed with 28-homobrassinolide, a brassinosteroid, showed improved resistance against the induced toxicity. Brassinosteroids are hormones that are reported to be involved in Cd-stress signaling and could thus be involved in the tolerance of plants to heavy metals [113] although its exact role remains to be clarified.

## 9. Conclusions

Even if we restrict our analysis to a few species within the genus *Brassica*, we observe variation in the published results regarding metal uptake and the defense mechanisms used by the plants, and most of the reasons for this have been discussed above. The variable experimental setups used in the experiments, as described in the beginning of this paper, can have a large impact on the observed results of the determinations under analysis.

There has been a large effort in the research of plants suitable for phytoremediation processes. Although some of the *Brassica* species are reported to be suitable for this environmentally attractive technique, and show a moderate to high tolerance to several heavy metals, it seems that its full potential has yet to be met. Cultivation of contaminated areas with tolerant species that could be used for energy production might be an attractive solution for the economically sound use of these land areas [114].

The chelating of heavy metals seems to be one of the most important mechanisms for the tolerance of *Brassica* species. A frequently described effect of metal toxicity is the increase in the levels of citrate and this seems to be an important factor in heavy metal tolerance in these plants, as this organic acid could be implicated in chelating the metals facilitating its translocation and accumulation in the shoots and reducing its toxicity. In favor of this conclusion is the fact that exogenous application of citrate to a contaminated medium not only improves the uptake of heavy metals but also improves plant tolerance. Phytochelatins are also widely considered to be very important in the resistance of certain *Brassica* species to heavy metal toxicity.

The role of heavy metal transporters is now much clearer in these plants and they seem to have a fundamental importance in the tolerance of *Brassica* species to different heavy metals.

Damage to *Brassica* plants is due to direct heavy metal effects or to induced oxidative stress. Frequently reported observations include stunted growth, reduced root growth and affected root morphology, affected photosynthetic activity and chlorosis, reduced uptake of water and of certain essential elements.

The advances in “omics” technologies in the last few years (including genomics, transcriptomics, proteomics and metabolomics) has allowed to start to characterize the genome, transcript, protein and metabolite levels involved in abiotic stresses like heavy metal stress [115], but there is still a long way ahead to enable the establishment of a clear picture of the tolerance and defense mechanisms used in *Brassica* species.

## Supplementary Materials

Supplementary materials can be found at http://www.mdpi.com/1422-0067/16/08/17975/s1.
